# Digital Twin Coaching for Physical Activities: A Survey

**DOI:** 10.3390/s20205936

**Published:** 2020-10-21

**Authors:** Rogelio Gámez Díaz, Qingtian Yu, Yezhe Ding, Fedwa Laamarti, Abdulmotaleb El Saddik

**Affiliations:** Multimedia Communications Research Laboratory, University of Ottawa, Ottawa, ON K1N6N5, Canada; qyu104@uottawa.ca (Q.Y.); yding058@uottawa.ca (Y.D.); flaam077@uottawa.ca (F.L.); elsaddik@uottawa.ca (A.E.S.)

**Keywords:** digital twin, smart coaching, artificial intelligence, machine learning, deep learning, fitness, obesity, sports, rehabilitation

## Abstract

Digital Twin technology has been rising in popularity thanks to the popularity of machine learning in the last decade. As the life expectancy of people around the world is increasing, so is the focus on physical activity to remain healthy especially in the current times where people are staying sedentary while in quarantine. This article aims to provide a survey on the field of Digital Twin technology focusing on machine learning and coaching techniques as they have not been explored yet. We also define what Digital Twin Coaching is and categorize the work done so far in terms of the objective of the physical activity. We also list common Digital Twin Coaching characteristics found in the articles reviewed in terms of concepts such as interactivity, privacy and security and also detail future perspectives in multimodal interaction and standardization, to name a few, that can guide researchers if they choose to work in this field. Finally, we provide a diagram for the Digital Twin Ecosystem showing the interaction between relevant entities and the information flow as well as an idealization of an ideal Digital Twin Ecosystem for team sports’ athlete tracking.

## 1. Introduction

The coach is one of the most influential figures in sports. Research has shown that a positive coach-athlete relationship enhances the athlete’s response to stress and improves his/her performance [1,2]. For the same reason, a negative coaching experience can severely affect the motivation and competence of the athlete [3,4]. Work is being done not only in training professional coaches but also in trying to make these professionals understand how to engage better with people who are not interested in sports [5]. However, not all people can have the luxury of having a trained professional to help them improve physically or overcome certain physical limitations.

This is where Smart Coaching comes into play as it can be a handy tool, if not a substitute for trained professionals, for people who cannot afford to attend a coaching session for one reason or another. Besides sports, Smart Coaching is also helpful for the aging population. The World Health Organization has declared the decade of 2020–2030 as the decade of healthy aging [6]. In their report on aging and health [7], they propose key areas of action to help healthy aging with one being “Ensure a sustainable and appropriately trained health workforce”.

The use of the Digital Twin technology can help bring a highly customizable solution to individuals. Digital Twin (DT) technology enables the collection and analysis of data about the real twin and provides them with personalized feedback to improve their quality of life and wellbeing [8,9]. The DT concept was first introduced in 2002 and referred to a virtual replication of a physical object. This concept was and is still highly beneficial in industry where companies, especially in the manufacturing industry, are adopting it massively due to the gains it brings when used in industrial processes.

The DT in industry refers to the comprehensive virtual replica of a product or process that is built using connected sensors and is based on mass historical data. This concept allows the building of optimal models and virtual testing at a much lower cost than when this is done on the actual physical product. In addition, the DT collects data in real-time and consequently represents the physical object at any given moment. This opens doors to predictions using the collected data as the artificial intelligence detects possible needs for maintenance before any breakdowns happen. The DT is very realistic and is supported by one or more interdependent phases including sensors selection, data acquisition, data analysis, model training, actuators program and iteration of results evaluation [10].

DTs were redefined in 2018 as a digital replication of living or nonliving physical entities [8]. This redefinition opened doors to using the potential of this promising concept for humans such as in the domain of health and wellbeing. Some analogies can be made between the industrial DT and the human DT and the concepts of industrial DTs can also be used in human DTs. For industrial DTs, it is important to consider the assessment and monitoring of manufacturing abnormalities that may affect the physical product, which can be, for example, a vehicle. The DT obtains sensor data from the vehicle’s integrated vehicle health management (IVHM) system, maintenance history and all available historical and fleet data obtained using data mining and text mining. Correspondingly, for human DTs, it is necessary to evaluate and monitor factors that may affect the human training process. The DT obtains sensor data from sensors such as the accelerometer/gyroscope, Electroencephalogram (EEG) and Electromyography (EMG) sensors, RGB Camera/RGB-D Camera, infrared Camera, Kinect, dynamometer, Heart Rate (HR) monitor and oxygen monitor. It then analyzes historical data and trains new data via deep learning and machine learning and produces recommender models. By merging all of the information above for both industrial DTs and human DTs, we can provide recommendations and predict to different extents future situations. By comparing the expected response with the actual response, DTs must make self-improvements, which makes the model more accurate and more applicable.

We suggest adopting the promising concept of human DTs in the domain of sport as well. Keeping a history of people’s training results over time helps in building a digital replica of their profile representing their DT. Furthermore, the smart training component augments this DT with coaching abilities, giving it the tools needed to coach the real twin to improve her or his performance. Every training session feeds the system with more and more data, which are then used to provide recommendations for improving the overall training experience. The DT of the trainee, augmented with Smart Coaching abilities, collects biophysical data and interacts in real-time with the real twin to provide better analysis and feedback.

**Figure 1:**
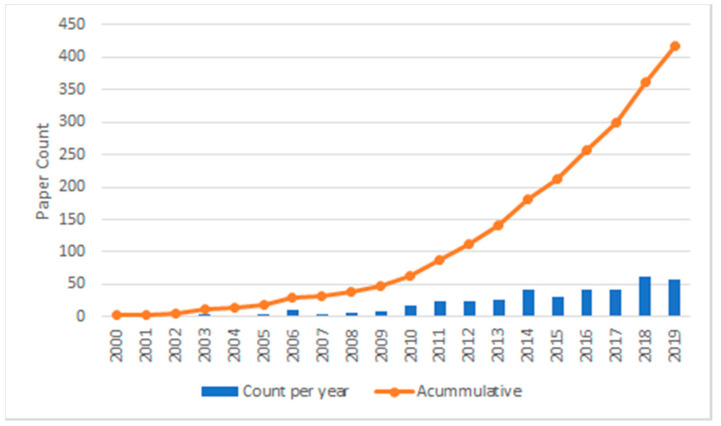
Hits for the concepts of “Smart Coach”, “Virtual Coach” and “Coach System” in the last 20 years in Scopus.

The concept of Smart Coaching has its roots from other fields such as e-learning and recommender systems. E-learning can be defined as “the learning supported by digital electronic tools and media” [11]. Therefore, we can consider the Smart Coaching component of the DT as a subset of e-learning, which has gained popularity in the last ten years. Students have become more and more used to a digital educational environment. They have a positive attitude towards e-learning [12] and online education is expected to become mainstream by 2025 [13], particularly after the COVID-19 era. The permeability of digital learning in the current population will help cement DT Coaching as a valid technology. In Figure 1, we can see the rising of Smart Coaching research in the last 20 years.

There is also a close relation between DT Coaching and recommender systems. A recommender system, in terms of e-commerce, is a tool that helps curate items from an extensive database of products or services to users based on their past preferences [14,15]. It makes a profile of the user and tries to predict the products that he or she may like. Both a DT Coach and a recommender system try to make a model of the user. User modeling is a research topic in Human-Computer Interaction focused on improving the user experience of systems by providing tailored experiences and recommendations to the user based on specific background knowledge and objectives [16].

DT Coaching is also made possible by all of the advances in hardware such as smart devices and sensors. In recent times, we take for granted the existence of smart devices all around us from our smartphone and smartwatch to smart lighting and other home appliances. In a 2019 article, Gartner reported that user spending in wearables in 2020 will reach USD 52 billion [17]. Smart devices are here to stay and with them smart technology is reaching the common masses with great success [18,19,20]. A DT Coaching System would not be possible without the advancements and ubiquity of such smart devices. Some characteristics of smart devices are “sensing and actuating”, “reasoning and learning” and “memory and status tracking” [21].

Probably the most popular tool in the arsenal of DT Coaches nowadays is machine learning (ML). It provides the intelligence and flexibility to DTs, adding a Smart Coaching component to the system. Machine learning has existed since the 1960s [22] but the current popularity of it is caused by the advances in computer processing and the availability of high amounts of data. It is forecasted that the machine learning market will reach USD 20.83 billion in 2024 [23].

In summary, our contribution includes:

- A definition of Digital Twin Coaching (DTC) that utilizes the potential of the Digital Twin to provide a custom service for users;
- A classification of literature based on our review into three parts that are Sports, Wellbeing and Rehabilitation. They are based on the target groups and their current and coveted physical condition;
- A summary of six required characteristics of a complete Smart Coaching System. Promising fields including Smart Equipment, Standardization, Effective Guidance and Integration of Haptics are put forward for future research;
- Future perspectives in the fields of smart equipment, standardization, pose estimation and multimodal interaction to guide future research in the topic of DT Coaching;
- An ecosystem of the Digital Twin Coaching, analyzing all of the actors involved as well as the modules that need to be working together to provide a good quality experience for both Coach and Trainee with an example applied to sports.

## 2. Definitions

### 2.1. Digital Twin (DT)

Digital Twins are defined as “digital replications of living as well as non-living entities that enable data to be seamlessly transmitted between the physical and virtual worlds” [8]. The use of a DT ranges from analyzing complex manufacturing processes using artificial intelligence to monitoring individual people and providing feedback to predicting possible health issues such as a heart attack before it happens [24].

A DT has several characteristics that are shared with Smart Coaching Systems; for example, the use of sensors for monitoring, having artificial intelligence as a core, being able to communicate in near real-time, having the means to interact with the real world such as actuators and be trusted by the users [25].

### 2.2. Smart Coach (SC)

Little to no work has been done in trying to define what a Smart Coach is but we can start by saying that it is a system whose objective is to coach people using many smart techniques. Using the Cambridge Dictionary website to define a “Smart Coach” (SC), we get the following:

- Smart: adjective (WORKING BY COMPUTER) “a smart machine, weapon, etc. uses computers to make it work so that it is able to act in an independent way.” [26];
- Coach: noun (TEACHER) “someone whose job is to teach people to improve at a sport, skill, or school subject.” [27].

Therefore, we can define an SC as a set of smart devices able to work independently with the objective of helping people to improve in a specific field.

### 2.3. Artificial Intelligence (AI)

Artificial intelligence (AI) is a concept of Computer Science that focuses on “the theory and development of computer systems able to perform tasks normally requiring human intelligence” [28]. It is the field of study of how to make computers “smart” and tries to enable a machine to simulate human behavior. It has many uses in automation, security and decision making, for example.

Artificial intelligence uses machine learning (ML) and deep learning (DL) algorithms to process data and discover relationships between the variables (input, output) from direct samples of a system. Deep learning has excelled in the field of computer vision over the last few years. DL has been used in tasks such as self-driving cars, designing bots for games such as Go or StarCraft, activity recognition and pose estimation.

### 2.4. Digital Twin Coaching (DTC)

We have defined the Digital Twin and Smart Coaching. For SCs to work independently, we need to collect data about the person in the specific field using several types of sensors. To achieve that, we can leverage the DT technology and form the person’s DT in the area of sport. An SC needs to then implement intelligence into the system. Using the capabilities of the Digital Twin, we can use artificial intelligence to provide highly customized and targeted feedback to the trainee.

DT Coaching can be defined as a Smart Coach that leverages the power of the Digital Twin technology to provide a tailored coaching experience to trainees. DT Coaching takes into account their current physical and mental condition with the help of Internet of Things (IoT) for fast and seamless communication between the Digital Twin and the real world.

## 3. Methodology

### 3.1. Research Questions

This survey aims at answering the following research questions:

1. What is the role of machine learning in physical activity digital coaching systems?
2. What physical activities benefit from the use of Digital Twin Coaching involving machine learning?

Therefore, we will be working with three main concepts that are Digital Twin Coaching, physical activity and machine learning.

### 3.2. Data Query and Extraction

To answer these research questions, we made queries about the concepts mentioned above in Scopus, Web of Science, IEEE Xplore and ACM Digital Library. We limited the papers to include the last ten years (2010–2020) to limit our work to the state-of-the-art.

The extraction of relevant information from the papers includes:

- ML algorithm(s) being used and why they were chosen;
- Type of application being researched;
- Sensors and actuators devices used;
- Performance of the ML algorithm;
- Usability feedback of users about the system.

## 4. Categorization

A categorization of the papers was needed to organize the results obtained from the methodology described above. Based on the literature reviewed, three categories stood out that were sports, wellbeing and rehabilitation. These categories depended on the target population and their current and desired physical state (Table 1).

In the case of sports, the user usually was in a good health state and wanted to improve their physical performance to be more competitive. In wellbeing, the person wanted to achieve good or better health habits and was not about being competitive but being healthy. The patients who needed rehabilitation sessions because of a physical impairment wished to recover their physical functions.

### 4.1. Wellbeing

Wellbeing is all about keeping a healthy life. The research work in this category is mainly focused on daily physical exercises such as stretching, spinning and yoga. Some papers focus on elderly care via corrective exercises and relaxation therapy. Most of these articles are about developing a gesture/pose recognition technology for the demands of exercise or elderly care with the help of wearable devices and cameras.

The research in [29,30] used different methods to realize elderly care. In [29], researchers designed a system in which a robot interacted with a person. This robot guided the person through several poses and checked if they performed those poses using a K-Nearest Neighbors (KNN) classifier. In [30], researchers designed a system that classified the position of a person as right or wrong using the Kinect sensor. First, they obtained the 3D skeleton of a person using the Kinect and then they passed this information to a support vector machine (SVM) that classified the position as right or wrong.

In terms of yoga, in [31], a 3-infrared camera system that obtained data from yoga postures and classified them in a server was designed. The authors in [32] developed a system in which they classified yoga postures using two Kinects. Researchers in [33] used the Kinect to recognize six yoga postures using Adaboost, which is an ensemble classifier that focuses on retraining the model several times using the incorrectly classified instances as the dataset [34,35]. In [36], the postures of a yoga teacher were collected using a Kinect and classified using a Nearest Neighbor (NN) for the students to follow.

For stretching, researchers in [37] used the Kinect to extract features from several exercises and classified them using Random Forests. In [38], the focus was muscle strength change and fatigue development during elbow extensors using a training protocol designed on a dynamometer and a non-linear model.

In [39], a coaching system was designed to be used for a wider array of exercise regimens by classifying large-scale exercise motion data obtained from a forearm-worn wearable sensor instead of recognizing a limited number of distinctive movements.

Kang et al. [40] developed a gesture recognition technology for spinning exercise in which the user used a spin bike to move the upper body to upper, lower, left, right and wave. They collected movement data from the user’s wrist and head using an inertial measurement unit (IMU) sensor then processed the information with a gesture recognition module and classified gestures into nine classes. They compared three different classifiers, which were statistical techniques, SVMs and decision trees. The decision tree classifier showed the highest gesture recognition rate. It was also reported that using bootstrapping improved the accuracy of the decision tree classifier.

There is also work done for sleeping patterns to improve wellbeing. In [41], researchers designed a system called Quantified Self in which they followed users for six months collecting information using wearables and medical sensors. The data was related to their sleeping quality and duration compared with the number of steps walked through the day. This information was then fed to a KNN algorithm.

### 4.2. Rehabilitation

Successful rehabilitation can be physically demanding for the person because it involves testing the body limits while trying to improve its physical condition. For rehabilitation, a professional coach involvement can be essential. This brings us to the same problem where sometimes there are not enough professionals available to coach every person who needs rehabilitation, be it because of high demand, inability to reach rehabilitation centers or cost, for example.

The target population of papers that focused on rehabilitation was different from those that focused on sports or wellbeing. This type of research is not focused on being competitive such as sports or on maintaining good health in daily life such as wellbeing. Rehabilitation papers focus on people who have lost motor ability and how to help them recover. They may have suffered a stroke, have movement disorders or their age has taken a toll on their bodies. The target population of the papers found using ML for rehabilitation is shown in Table 2.

Motor imagery of an electroencephalogram (EEG) is different in people who have had a stroke due to injury in the motor cortex. Therefore, traditional methods of motor classification of an EEG would not work [42]. One way to solve this is proposed in [42] where they used F-boost to improve a set of weak common spatial pattern (CSP) SVM classifiers. The reasoning behind using boosting was that CSP-SVM classifiers worked well with healthy subjects, which was not the case in stroke patients for the reasons stated above. In [47], they tried an iterative algorithm to improve these weak CSP-SVM models that classify EEG data as a left hand and right hand movement.

An electroencephalogram (EEG) is a non-invasive brain imaging technique that uses scalp electrodes to measure the voltage fluctuations induced by the mass electrical activity of neurons [55]. An electromyography (EMG) technique is usually used to record the electrical activity produced by skeletal muscles [56].

Using EEG and EMG, researchers in [45] were able to classify not only motion but also fatigue using one SVM algorithm for each task with an accuracy of 99% in healthy subjects and 95% on sick subjects for upper limb rehabilitation.

In [44], they also worked with upper limb rehabilitation using the Kinect. Researchers tasked the subjects to perform several motor imagery tasks while their movement was being measured in three aspects that were precision, smoothness and compensation.

Furthermore, the work by Liang et al. [43] focused on lower limb rehabilitation using a Kinect and 3-axis accelerometer-gyroscope combination. They proposed a system that created a gait model of the patient for it to be used in conjunction with an exoskeleton to guide the person in their rehabilitation sessions. Researchers have also proposed a mechanical feedback system for upper limb rehabilitation [46]. They used surface electromyography (sEMG) to classify the motion of the unaffected hand and had the hand exoskeleton designed to perform the same movement on the affected hand. Upper limb rehabilitation research was also proposed in [51] where researchers trained a model to predict wrist motion using EMG sensors (MyoWare sensors) to move an exoskeleton robot.

Another exciting hand motion feedback approach was proposed in [49]. Here, researchers had the subjects performing tasks in a virtual reality (VR) environment. They designed a kitchen in the VR environment and had the users perform tasks such as cracking nuts. For classification, they also used sEMG and an SVM. In [48], researchers worked with hand, wrist and forearm movements. Using Linear Discrimination Analysis and an SVM, they found that wrist accuracy was the highest of the three motion tasks while hand accuracy was the lowest.

Going to another target demographic, we found the work of Wang et al. [52]. They had children with Cerebral Palsy walking on a treadmill while they recorded their sEMG. The objective of this study was the classification of gait phases using an SVM. In [53], researchers used OpenPose [57] to extract the features of the movement of specific rehabilitation exercises and the collected information was then compared with the standard GMFM-66 [58] to produce a score. This could then be used by professionals to help in the rehabilitation process even if they were not in the same place.

### 4.3. Sports

Sport is a competitive field that demands a high athletic level. It requires professional training, which includes pose analysis, pose correction, reasonable training plans and timely fatigue control. However, not all athletes are equipped with an expert and sometimes athletes may need to train alone. Smart Coaching is an excellent way to help users make progress and achieve training goals that meet the requirements of athletes perfectly.

Most systems in sports take advantage of machine learning algorithms to give professional pose corrections or recognition to players in many fields such as baseball, biking, skiing, Tai Chi, swimming, weightlifting and tennis. The whole process includes gathering body information from sensors, feeding the data into ML models and giving the recognition and classification results.

Kamel et al. [59], created a Convolutional Neural Network (CNN) based system that compared the student pose with a Tai Chi teacher that provided real-time feedback via a user interface. The feedback included motion replays of the user’s 3D skeleton model compared with the template model and a score report. The authors in [60] trained a Tai Chi coaching system using an SVM with features extracted from Random Forests. Besides providing real-time feedback on the trainee’s avatar, the system also used a Cave Automatic Virtual Environment (CAVE) based sports training environment.

In the swimming field, researchers have worked on swimming section division (stroke and turn) and style classification using linear regression [61] and decision trees [62] with data collected by a waterproof sensor, which included a built-in acceleration sensor and a velocity sensor. The reasoning behind it was that those sensors did not suffer from water turbulence and unclear motion under water compared with camera-based sensors.

In weightlifting, Yasser et al. [63] detected the misplaced joints of the athletes while lifting. It compared different models to see which one was more accurate in classifying correct postures in fundamental lifts such as deadlifts, shoulder presses and squats. Other researchers [64] proposed a deep key frame extraction method for analyzing weightlifting videos and focused on pose probability of each frame.

In the area of tennis, researchers have designed an autonomous Echo State Network (ESN) ensemble model for temporal phasing of forehand swings to achieve better results compared with a single optimized ESN model [65]. Bačić et al. [66] have demonstrated a radial basis function (RBF) SVM based classifier to identify tennis swings automatically. They were able to estimate swing techniques based on different criteria given by the coach. In table tennis, Wu et al. [67] gathered the upper body information by a CNN model. They then trained an LSTM (long short-term memory) model to predict the ball movement using an ordinary camera to track the ball movement even before serving.

For golf, Zhang et al. [68] designed a system that analyzed the poses and scores, which was based on an SVM and GMM (Gaussian mixture model).

In [69], researchers developed a martial art training system based on real-time human pose forecasting. It used recurrent networks (LSTM) to learn the temporal features of the human motion and passed them to the 3D recovery network. They used VR to show the predicted movements of the trainees.

Some researchers also tried to apply the model for all of the videos of exercise. In [70], a system proposed a system for videos of sports, which largely depended on the correctness of human poses. The system included trajectory extraction, human pose estimation and pose correctness. They achieved good results in tracking, pose estimation using other sports datasets such as VOT2018-LT and Penn Action. In the pose correctness part they tested using their dataset, which mainly consisted of skiing videos. They also did a usability test, which compared their apps with other applications such as Keep. It is promising because it can be applied to many other sports.

There are also a few other interesting applications in the sports field. Researchers in [71] designed an NN to create a training plan for cyclists based on power meter data and compared it with human-made programs from the platform TrainingPeaks. There is also work done to recognize referees’ gestures in real-time using a supervised/semi-supervised hybrid NN [72].

## 5. Technical Attributes

### 5.1. Algorithms

As we can see in Table 3, an SVM was the most used model and widely applied in different fields. In the rehabilitation field, an SVM was commonly adopted in analyzing signals inside the human body. Researchers in [42,43,49] used CSP for feature extraction and applied an SVM to classify EEG data for two-classes motor imagery classification and hand and wrist movement classification. Others adopted an SVM to handle EMG signals for gesture, gait phase and wrist position recognition [48,50,51]. Wang et al. [52] applied an SVM to both the EMG and EEG signals for predicting the users’ intention and fatigue. One exception in this field is the work done by Ukita et al. [47], which used an SVM for pose classification and mining crucial features such as position and velocities using Kinect data.

In the sports and wellbeing field, an SVM is not as popular as in the rehabilitation field. An SVM was applied to detect trainees’ errors using data from sports videos [60]. In paper [30,68], they utilized an SVM to analyze position and motion from Kinect data. Researchers also classified gestures using data from wearable devices utilizing an SVM [40].

In addition to SVMs, models based on neural networks are popular as well with CNN applied more frequently. Some researchers also use Recurrent Neural Networks (RNN), Echo Stat Neural Networks (ESN), Wavelet Neural Networks (WNN) and Long-Short Term Memory Neural Networks (LSTM) in their work. CNN is competent to extract features especially in time-series data classification due to the advantage of scale invariance and local dependency. Most neural network models are applied in the wellbeing and sports fields for pose or gesture estimation. Parmar et al. [73] used neural networks to score the Olympic games, which was also related to pose estimation. They used NNs because they should process the whole video to recognize or classify the poses. Other applications included designing a training plan [71] where it needed to retain some partition of the user’s past performance, so an RNN based model was applied.

Many papers apply distance-based models such as KNN. They mainly use it as a classifier in sports fields; for example, to classify tennis swings [66], to recognize jumps and evaluate the movement intensity [74] and to guide users’ exercises pose [29] in rehabilitation.

Five models included decision trees and random forests. The work in [60] used random forests to select features for further use. In other cases, they were used as classifiers [40,62,64]. Regression models were also discussed including linear regression [38,43,61], support vector regression [43] and Gaussian process regression [43]. Besides these popular algorithms, other algorithms such as echo state networks (ESN) [65], wavelet neural networks (WNN) [44], fully convolutional neural networks (FCN) [64] and Bayes’ theorem [63] were also used.

### 5.2. Sensors

As Table 4 shows, Kinect was undoubtedly the most popular sensor due to its ability to capture 3D depth information of the player and the environment, making the processing much more manageable. In a few cases where users needed speed and orientation information, they chose to use wearable sensors with accelerometers or gyroscopes inside. In the rehabilitation field, EEG and EMG were popular due to the demand for accuracy in this field. In addition, systems using RGB cameras and markers were used for capturing human pose information. In some other papers, dynamometers were used to calculate energy consumption.

### 5.3. Tools/Platform

**Figure 2:**
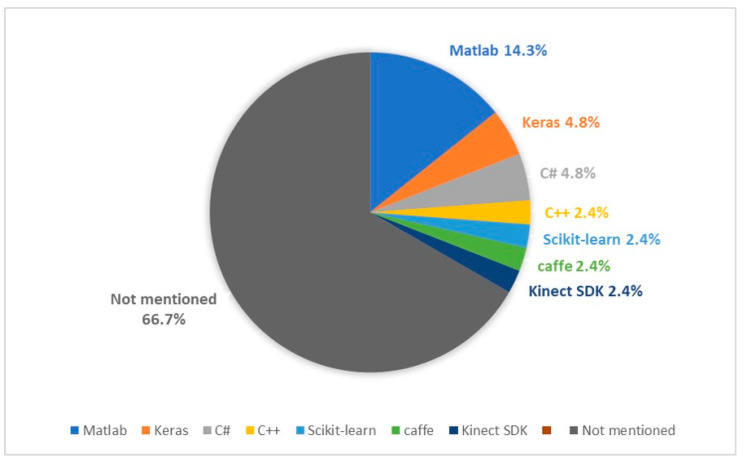
Breakdown of platforms and tools used.

More than 65% of papers did not reference any kind of tool or platform for their development (Figure 2). Six articles used MATLAB code. C# and Keras were each used in two papers. C++, scikit-learn, CAFFE and Kinect Software Development Kit (SDK) were also discussed in other works (Table 5).

## 6. Discussion

### 6.1. Characteristics of a DTC System

We found common characteristics in many of the systems surveyed in this paper, which we also compared with the literature in the related fields of recommender systems, e-learning and coaching. Therefore, we found it relevant to list the required characteristics of a complete Smart Coaching System, which were collected in this survey (ordered alphabetically).

#### 6.1.1. Auditability

The system should be able to have quantitative or qualitative usability measurements on how it is helping or how it can help the user improve [75,76,77]. In other words, the coaching systems should be able to provide a way to prove they work such as via standardized usability tests such as the System Usability Scale [78]. However, there is work to be done here as a standard to audit digital coaching does not exist. There is an evident gap in how to assess a DTC’s usability correctly. As these types of systems are a combination of many fields such as physical exercise coaching, IoT devices and AI, it is difficult to accurately determine the correct way to judge the interaction interface and the feedback of the user.

#### 6.1.2. Autonomy

DTC should be supplied with all of the input from professionals that is necessary for it to provide coaching to users. Once trained, the DTC should be able to work independently without extra input from professionals as this is an inherent ability of any kind of smart device/system [79].

#### 6.1.3. Credibility

The DTC should be trained with the help of professionals or using professional-grade techniques and the user should be aware of this. The users need to trust the system as the relationship of coach-athlete is of utmost importance [1,2]. To improve the credibility of DTC, researchers should involve trained professionals in their work. It is of utmost importance to have a professional’s feedback when designing DTC as it can help ensure that the system meets the criterion of successful coaching and avoids any negative consequences that could be caused by the system such as injuries. Furthermore, it would be useful to also get feedback from coaches as this can help discover new areas of opportunities in the system.

#### 6.1.4. Flexibility

As the user performance changes over time, the DTC should be able to account for this and make the necessary changes to accommodate best the user’s skill [80,81]. There is still a lot of work to be done in this field as we could see in the requirements of DTC. Researchers can work on a way to integrate the trainee’s past performance in the intelligence module of the system. This way, the Smart Coach can also learn from his or her behavior to give better feedback and, if possible, predict future performance to motivate the trainee.

It would also be promising to save the training history such as exercise time, exercise duration and rest duration. It would help not only to have the necessary information to remind the trainee to finish the planned training but also to provide information on the user’s body condition including athletic ability and possible fatigue level when training or performing rehabilitation. This data can also be analyzed by the coaching system to revise the upcoming planned training sessions.

#### 6.1.5. Interactivity

DTC should be able to collect information on the users’ performance using different kinds of sensors. The system needs to give feedback on the users’ current performance, how to improve and how they have been doing through the coaching sessions [79].

#### 6.1.6. Privacy and Security

When working with personal data, it is of importance to ensure it is securely stored or transmitted and that the privacy of the user is respected. Almost none of the papers in this survey explicitly mentioned this topic even though privacy is becoming more and more concerning in recent times. Being able to assure the users that their data are handled securely especially when working with medical data in the field of rehabilitation, for example, is a priority. We want the users to trust the DTC as they would put their trust in a professional coach.

### 6.2. Adherence of Research Work to the Derived Requirements

Table 6, Table 7 and Table 8 show how the surveyed papers adhered to these requirements. We can see that most of them followed between one and three of these requirements; only one paper considered the security requirement and very few articles did not follow any of the derived requirements.

### 6.3. DT Coaching Ecosystem

Establishing an ecosystem for Digital Twin Coaching is important because we can see all of the actors and modules involved as well as how the data flows through the system. In [25], El Saddik et al. proposed a general ecosystem for Digital Twin for healthcare that involved seven main parts that were the data source, an AI-inference engine, multimodal interaction, cybersecurity, privacy, communication and a feedback loop. In [82], researchers focused on implementing the DT ecosystem for coaching for Edge Computing. They included a second actor in the ecosystem that was the coach. The coach provided another source of data that interacted in a similar way as the main actor, which was the trainee.

**Figure 3:**
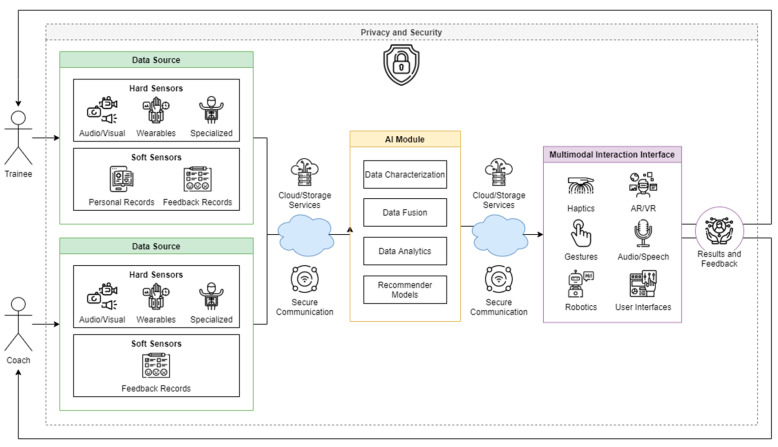
DT Coaching Ecosystem adapted from [25,82]. Icons from flaticon.

In Figure 3, we can see an adapted ecosystem for DT Coaching. We have the two actors; the trainee and the coach. These actors have two types of sensors that are hard and soft. Hard sensors in this case are devices that collect biophysical data such as heart rate, oxygen levels and video feed. Soft sensors are virtual and they collect information such as personal and medical records or instant feedback data entered in form of questionnaires, for example. The coaches do not need to have their personal record information collected as the system is not trying to adapt to them, but to the trainee.

The smart module is the one in charge of the processing of all of the information as well as selecting relevant information to save and present to the actors. Here we can find ML and DL algorithms as well as recommender models providing the intelligence to the system.

A multimodal interaction interface makes use of all of the hardware and software that the DT Coaching ecosystem has access to in order to interact with the different actors from the simplest user interfaces to more complex solutions such as haptics enhanced AR/VR.

We also have all of the middle interfaces and services that glue the system together such as cloud and storage services for data backup and communication channels that can be wired, Wi-Fi, Bluetooth or cellphone data, for example.

Finally, we have two of the most important elements in the ecosystem, which are privacy and security. These two elements englobe all of the stages from the simplest data collection to the way it is presented to the actors after being processed by the smart module. Without privacy or security, the whole ecosystem crumbles down.

### 6.4. Future Perspectives

The characteristics mentioned above are ones that a promising DTC should achieve given the advantages and functions they provide to the user. Therefore, in future research work, it is essential to consider them. In addition, analyzing the surveyed papers also allowed us to derive promising areas of open research, which we discuss in this section.

#### 6.4.1. Leveraging Smart Equipment

Smart physical activity devices equipped with sensors are an essential part of DTC providing easy access to trainees’ real-time data. This equipment should have sensors, microprocessors, a communication unit and a power source. Other than the use of a camera, not many other sensors such as wearable devices or other smart equipment were used in the current research work. We provide here examples we found in literature, which show a promising use of such equipment in future research.

Let us take smart insoles as an example. A group of researchers from RMIT University developed an insole with piezoelectric transducers to measure and analyze gait when kicking a soccer ball, which could collect trainees’ real-time gait information and help make an effective coaching plan [83]. In [84], researchers designed an insole that embedded a grid of 64 pressure sensing elements that use light emitting diodes (LED) and light sensor pairs to measure pressure distribution and the center of pressure. A shoe insole designed in [85] consisted of 12 embedded FSR (force-sensitive resistor) sensors and an IMU sensor. Its circuit was printed on a 2-layer flexible Printed Circuit Board (PCB) to enhance durability and quality. These insoles could be used to accurately detect trainees’ sole pressure distribution in any activity such as walking, sitting and running, which is useful in sports, wellbeing and rehabilitation. They could be used by the DTC to gather information and help fix any issues the real twin may have in his/her standing behavior such as posture.

There is also the smart vest introduced in [86]. It consists of a temperature sensor, ECG electrodes, ECG belts, woven wires, PPG sensors, GSR electrodes and a wearable data acquisition system designed using a microcontroller with wireless communication and global positioning system (GPS) modules. It can detect the wearer’s physiological signals in different parts of the body that offer a multi-source input to the DTC. The more biometric information DTC can have, the more accurate and efficient the coaching will be.

In [87], researchers designed a chair called “Aachen SmartChair”, which was integrated with a measurement system that obtained an ECG by a wireless communication module without direct skin contact. The chair offered an available platform for the quick detection of trainees’ ECG information, which is helpful especially when talking about DTC for rehabilitation because it can be used as an interface for people with disabilities.

#### 6.4.2. Standardization

An important area to take into consideration in research works is the standardization to allow plug and play interoperability for the DTC. Interoperability should be an intrinsic feature of the DTC as it allows it to join forces with other existing systems. One way to do this is by implementing protocols such as the X73-PHD as proposed in [88] and applying them to coaching systems. The X73-PHD set of standards provides a standardized communication protocol for personal health devices such as the ECG, pulse oximeters and weighing scales. This standard defines the communication protocol between personal health devices and managers, which can be a server, a PC or a mobile phone.

Following the protocol described above, we can implement it in any other smart equipment device. The advantage of having a single protocol for all communicating agents (sensors, actuators, servers, etc.) is that it can provide a solid infrastructure ready for IoT, which helps in the implementation of DTC.

#### 6.4.3. Effective Guidance

Effective guidance is a key to DTC and many systems focus on helping trainees with pose estimation and correction such as AI Coach [70], which detects wrong poses based on video input and presents related video examples of 2D poses for athletes. Another coaching system in [47] shows a 2D projection motion comparison, 2D/3D joint distance and joint speed deviation. Tai Chi [59] gives motion replay, marks the wrong joints with yellow and provides a score based on the overall performance.

The challenge here is that it is inevitable to have areas in the video where body parts overlap. In this case, a 3D pose is more suitable in posture guidance to meet the requirements of displaying body information. Users can rotate the 3D model of the coach to get a better view perspective in case occlusion happens. For instance, most coaching systems provide a side view of coaches while training planks. Trainees may not know the distance between two feet or two elbows without further explanation and with 3D pose guidance, users can change the view from side view to top view to get this information.

Nevertheless, both 3D pose estimation and classification are complicated issues and are often accompanied by high deployment costs. Complex machine learning models such as deep neural networks make it hard to be integrated into devices with limited computing power such as mobile phones and embedded systems. One solution is building the 3D pose prediction model based on light models such as MobileNet [89]. In short, MobileNet is a neural network that focuses on reducing the size of the model and complexity, facilitating the implementation in smart devices such as wearables.

Another solution is Teacher-Student learning [90]. This methodology is based on knowledge distillation, which transfers knowledge between two different networks. Models tailored for smart devices are small and hard to train. In this case, large networks (Teacher networks) can be trained first and knowledge is then transferred to the required small network (Student networks) [91].

#### 6.4.4. Multimodal Interaction: Integration of Haptics

The fourth promising research area is concerned with the gap in haptic interaction. Most coaching systems surveyed give trainees audio or visual feedback, mainly following one of these two scenarios. One is to demonstrate the scores of recognition accuracy or to show visual results on a screen. The other is to remind users by audio instructions. There is indeed a lack of haptic feedback in current coaching systems.

Haptic interaction, including both tactile and kinesthetic senses such as vibrations, forces and motion, can easily provide a better training experience and simulate a real coaching environment for trainees [92]. In order to establish a haptic interface that allows a user to interact with the DTC, multiple actuators can be used in key body parts and according to different coaching plans. An actuator is a device that takes as an input a control command and outputs a change in the physical environment such as vibrators inside smart clothes [93].

Some concepts such as VR, AR and MR have been successfully introduced to coaching systems. Haptic rendering, which refers to the process of computing and generating forces in response to user interactions with virtual objects, can be a practical way for a trainee to improve interaction with the DTC. Haptic rendering enables a user to touch, feel and manipulate virtual objects through a haptic interface [94].

### 6.5. DT Coach Idealization Concept: DTCoach

**Figure 4:**
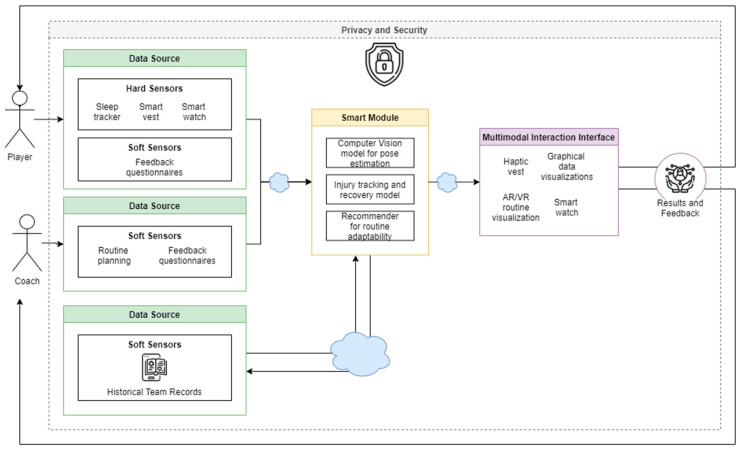
DTCoach System.

After discussing the DTC characteristics, ecosystem and future perspectives, we idealize an example of a complete DT Coach called “DTCoach: A Digital Twin Coaching System for Sports” (Figure 4). In this DT system we have the two main actors, the Player and Coach. The objective of this system is to have the DT of a Player interact with the Coach so it can enhance the overall training performance.

The Player data are collected using several devices such as a smart vest [95] that can be used as a sensor and haptic actuator, sleep tracker and smartwatch, for example. This information is then collected by the artificial intelligence (AI) module. The AI module keeps track of the athlete or the trainee in general and extracts activities from the sensed data. With the help of a recommender model, we can tailor each training routine for each player to avoid injury [96]. We can also use cameras to track the athlete’s movements [63] so that we can correct mistakes in the exercises.

The AI module also plans the training routine and communicates it to the athlete using the multimodal interaction interface of the DTCoach system, which also shows the improvement over time of the athlete using graphical visualizations. The athlete can also get real-time feedback using haptic enabled devices such as the smart vest discussed above or wearables such as smartwatches.

## 7. Conclusions

Technology has been used to enhance performance in different fields and coaching can certainly benefit from it as well. The last decade has seen the exponential growth of smart technology such as smartphones and smartwatches in parallel with the current interest in developing new and better AI algorithms. The advances in these two domains can make Smart Coaching available to the general population.

DT Coaching is a relatively new field that combines several others such as machine learning and human-computer interaction. Therefore, research is still trying to figure out which aspects are essential and which are not. In this survey, we first divided DT Coaching research articles into three categories depending on the objective of the coaching system that were wellbeing, sports and rehabilitation. We also summarized which algorithms, sensors, tools and platforms were used. In addition to that, we provided a list of common characteristics found in the articles reviewed. These future perspectives could be useful to future researchers in their new endeavors related to Digital Twin Coaching.

Researchers need to consider the different aspects of a Smart Coach, especially if they want to take it to the next level of design of DTC. Some of these characteristics such as privacy and security in the field of DT Coaching have a lot of room to grow as current articles indicate that researchers put little focus on them. There are also several fields that, in tandem with DT Coaching, can provide for a lot of exciting research paths. We can enhance the DT data collection and interaction using complex smart equipment, use standards that help the DT communicate with other DTs and technologies and include haptic actuators into our systems. All of these technologies can enrich the trainee’s experience with the DT Coach, to name just a few possibilities.

## Figures and Tables

**Table 1:** Comparison of categories.

Category	State of the Person	Objective
Sports	Good health	Competitiveness
Wellbeing	Good/bad health	Achieve/maintain health
Rehabilitation	Poor health	Recover health

**Table 2:** Target population of research on rehabilitation.

Target Population	Articles
Stroke patients	[42,43,44,45,46,47,48,49,50,51]
Cerebral palsy patients	[52,53]
Other	[54]

**Table 3:** Algorithms used in the articles reviewed.

Algorithm	Articles
SVM	[30,40,42,43,47,48,49,50,51,52,60,68]
CNN	[31,39,53,59,64,67,69,70,72,73]
KNN	[29,41,51,63,74]
Trees	[37,40,44,60,62]
RNN	[71,72,73]
Linear regression	[43,51,61]
GMM	[50,68]
NN	[36]
LSTM	[67,69]
ESN	[65]
WNN	[46]
Non-linear regression	[38]
FCN	[64]
Bayes	[63]

**Table 4:** Sensor types in articles reviewed.

Sensor	Articles
Kinect	[30,32,33,36,37,43,44,50,54,63,68]
Accelerometer/Gyroscope	[39,40,43,61,62,72,74]
EEG/EMG	[42,45,46,47,48,49,51,52]
RGB Camera	[53,60,67,70]
RGB-D Camera	[29,59]
Dynamometer	[38,71]
Infrared Camera	[31]
HR and oxygen monitor	[41,74]
Glucometer	[41]

**Table 5:** Platforms and tools used in articles reviewed.

Platform/Tool	Articles
MATLAB	[48,49,50,65,66,74]
Keras	[31,60]
C#	[32,33]
C++	[52]
scikit-learn	[60]
CAFFE	[73]
Kinect SDK	[33]

**Table 6:** Rehabilitation articles.

Article	Flexibility	Auditability	Autonomy	Credibility	Interactivity	Security and Privacy
[42]			✔		✔	
[43]						
[44]			✔	✔	✔	
[45]			✔		✔	
[46]			✔		✔	
[47]			✔		✔	
[48]			✔		✔	
[49]			✔		✔	
[50]			✔		✔	
[51]			✔			
[52]			✔			
[53]			✔			
[54]			✔			

**Table 7:** Sports articles.

Article	Flexibility	Auditability	Autonomy	Credibility	Interactivity	Security and Privacy
[59]		✔	✔		✔	
[60]			✔	✔	✔	
[61]			✔			
[62]			✔			
[63]			✔			
[64]						
[65]						
[66]			✔			
[67]		✔	✔			
[68]			✔			
[69]		✔	✔		✔	
[70]		✔	✔		✔	
[71]	✔		✔			
[72]			✔			
[73]			✔			
[74]	✔	✔	✔			

**Table 8:** Wellbeing articles.

Article	Flexibility	Auditability	Autonomy	Credibility	Interactivity	Security and Privacy
[29]			✔		✔	
[30]			✔		✔	
[31]			✔		✔	✔
[32]			✔			
[33]			✔		✔	
[36]			✔	✔	✔	
[37]			✔		✔	
[38]						
[39]			✔			
[40]			✔		✔	
[41]	✔		✔		✔	
